# Histone H2A.Z deregulation in prostate cancer. Cause or effect?

**DOI:** 10.1007/s10555-013-9486-9

**Published:** 2014-01-08

**Authors:** Deanna Dryhurst, Juan Ausió

**Affiliations:** 1 Department of Biochemistry and Microbiology, University of Victoria, Petch building, 258a, Victoria, British Columbia Canada V8W 3P6; 2 ImmunoPrecise Antibodies Ltd., 3204-4464 Markham St., Victoria, British Columbia Canada V8Z 7X8

**Keywords:** Histone variants, Prostate cancer, Androgen independence

## Abstract

Genetic and epigenetic changes are at the root of all cancers. The epigenetic component involves alterations of the post-synthetic modifications of DNA (methylation) and histones (histone posttranslational modifications, PTMs) as well as of those of their molecular “writers,” “readers,” and “erasers.” Noncoding RNAs (ncRNA) can also play a role. Here, we focus on the involvement of histone alterations in cancer, in particular that of the histone variant H2A.Z in the etiology of prostate cancer. The structural mechanisms putatively responsible for the contribution of H2A.Z to oncogenic gene expression programs are first described, followed by what is currently known about the involvement of this histone variant in the regulation of androgen receptor regulated gene expression. The implications of this and their relevance to oncogene deregulation in different stages of prostate cancer, including the progression toward androgen independence, are discussed. This review underscores the increasing awareness of the epigenetic contribution of histone variants to oncogenic progression.

## Introduction

Prostate cancer is the fifth most commonly diagnosed cancer worldwide [1] and the second leading cause of cancer-related death in men in the USA [2]. Crucial to the molecular etiology of this male cancer is the androgen receptor (AR), a member of the nuclear receptor superfamily. AR is present in the cytoplasm in its inactive form. Upon interaction with androgen, it undergoes a conformational change that allows it to homodimerize prior to binding to the DNA androgen response elements (AREs) [3]. AREs consist of inverted hexameric DNA half-site-like recognition sequences (5′-TGTTCT-3′) spaced by 3 bp [4] that are localized within the promoter and enhancer regions of AR-regulated genes. The ligand bound AR recruits co-activator proteins, including chromatin remodeling complexes that have histone posttranslational modification (PTM) activity, as well as components of the basic transcriptional machinery leading to gene expression. One such co-activator is SNF2-related CBP activator protein (SRCAP) that catalyzes the ATP-dependent incorporation of H2A.Z–H2B heterodimers into chromatin [5–7] and whose deregulation plays a critical role in prostate cancer [8].

Histone H2A.Z is a replacement histone variant that is expressed throughout the entire cell cycle in a replication independent way [9]. It is so far the only histone that has been shown to be indispensable for survival in many organisms and while only a single gene copy is present in invertebrates, two distinct gene copies exist in vertebrates [10]. These encode two functionally different H2A.Z-1 and H2A.Z-2 subtypes [10, 11] that have recently been implicated in prostate cancer [12].

As histone variants come into the limelight of epigenetic regulation in cancer [13], this review discusses different aspects of one of the most structurally and functionally controversial variants, H2A.Z [9], and its involvement in prostate cancer.

## Histones and cancer

Although many cancers have a genetic origin, most of them are either additionally subject to or are the direct result of epigenetic alterations as well. The role played by external factors such as the environment, smoking, life style, and viral infection in cancer etiology, and progression have long been recognized.

For many years, the molecular mechanisms underlying such epigenetic effects were ascribed to alterations in the pattern of DNA methylation [14, 15]. Only more recently has the role of histone PTMs and histone variants started to be fully appreciated [16, 17]. Histones can change the genomic landscape through two main types of mechanisms: histone PTMs (such as for instance methylation) and replacement histone variants (see below). In contrast to DNA that can only be methylated and hydroxymethylated, histones have a wider spectrum of PTMs and are present in all eukaryotic cells and organisms from sponges to humans. DNA methylation is highly restricted to certain metazoan groups [18]. Therefore, it is not surprising that histones have taken the main stage of epigenetic research.

The quest for a potential relationship between histones and cancer started long ago. In 1954, it was observed that histones from cancer cells exhibited an altered solubility and electrophoretic mobility [19]. Although the nature of these altered properties remains somewhat unexplained, it nevertheless pointed to the idea that histones from cancer cells can differ from those from nonmalignant cells. The most recent demonstration of this idea is from the observation that histone mutations drive pediatric glioblastoma [20]. Also, the link between alterations in histone PTMs and cancer is now very strong and experimentally well-documented. For instance, loss of H4K16Ac and H4K20Me3 have been shown to be general hallmarks of human cancer [21] and changes in the levels of global histone PTMs can be used as predictors of prostate cancer recurrence outcomes [22].

## Epigenetic mechanisms of histones

From a structural point of view, histones can be classified into core (H2A, H2B, H3, and H4) and linker histones (histones of the H1 family). The former are responsible for wrapping the DNA around an octameric histone core leading to the organization of the basic subunit of chromatin; the nucleosome core particle. The latter bind to the linker regions connecting adjacent nucleosomes and further contribute to the folding of the chromatin fiber. From a more functional perspective, histones can be essentially classified into two major groups: replication dependent (canonical histones) and replication independent (histone variants) [9]. Replication dependent histones are present in gene clusters [23] and are massively expressed during S-phase of the cell cycle. They provide one of the best examples of eukaryotic genes whose transcripts lack polyadenylated tails, and they lack introns. With the advent of new powerful spectrometry approaches, a plethora of canonical histone variant paralogs [24] have been identified in different cells and tissues that differ among themselves by only one or two amino acids (see for instance [25]). In contrast, replication independent histones are expressed at much lower rates throughout the entire cell cycle. Their individual single copy genes are at isolated positions on the chromosomes, their mRNAs are polyadenylated, and they contain introns [23]. They can replace the canonical histones and are usually referred to as replacement histone variants.

In addition to their own structural variability, all histones are amenable to a large variety of PTMs. To date, at least 15 different kinds of physiologically relevant histone PTMs have been described including: acetylation [26, 27], methylation [27, 28], phosphorylation [29, 30], poly-ADP ribosylation [31], ubiquitination [32], sumoylation [33], prolyl-isomerization [34], glycosylation [35], glycation and oxidation [36], crotonylation [37], biotynylation [38], succinylation [39], malonylation [39], lysine deamination [40], and glutathionylation [41]. Alterations of these histone marks can have dire effects on development and are associated with many varieties of cancers, including prostate cancer [42] (see [43] for a database resource of epigenetic marks in prostate cancer).

Histone PTMs can be classified into global long-range [44] and specific PTMs. Long-range PTMs are widespread over long genomic regions and often have important structural effects of their own [44], whereas specific PTMs take place locally at specific regions of genes (promoters and enhancers) and can be present in a combinatorial fashion creating a “code” [45] that can be deciphered by downstream effector protein complexes that either repress or enhance transcription.

The concept of histones and their PTMs having an epigenetic role and its potential involvement in carcinogenesis was realized in the early 1970s [46, 47], but its full significance was not appreciated until more recently. It has its roots in the so-called “histone code” hypothesis [45] according to which, specific histone PTMs by themselves or in a combinatorial fashion, can operate as marks that can be “read” by transcriptional effectors that regulate gene expression. How these histone PTMs are inherited throughout cell division and hence are true epigenetic marks are still a matter of controversy. Yet, histone PTMs determine the chromatin landscape that differentiates one tissue type from another and are critical for development of the organism. This makes it easy to understand the important implications that deregulation of such signals may have in cancer [48]. Although initially ascribed to histone PTMs, the “code” notion can be made inclusive of histone variants which constitute an additional combinatorial layer of complexity to histone epigenetics [9, 49, 50]. Like with histone PTMs, changes in the histone variant composition have been shown to play a role in cancer [13, 51–53]. Alterations in the levels of expression of linker histone variants [54–56] and core histone variants (H2A.1 and H2A.2 [57–61], H2A.X [62, 63], and macro H2A [64, 65]) have been observed in several types of cancers. Of particular interest is the recently described potential involvement of histone H2A.Z in cellular proliferation [66] as it pertains to prostate cancer progression and prognosis [8, 12, 67, 68].

## Histone H2A.Z: two subtypes and a controversial role

Histone H2A.Z is a replacement variant that has an ancient origin in the evolution of the histone H2A family [69, 70]. It is present in yeast, and it is encoded by a single gene throughout the invertebrate phyla [10]. Likely as a result of the whole genome duplication that took place at the onset of vertebrate evolution, it diversified into two genes with different promoters, 5’ UTRs and intron/exon organization [10]. Such an event probably led to a subfunctionalization of the encoded proteins (H2A.Z-1 and H2A.Z-2) that differ by only three amino acids [10, 11]. With the exception of yeast, H2A.Z has been shown to be indispensable for survival in several model organisms including the ciliate protozoan *Tetrahymena* [71], *Drosophila* [72], *Xenopus* [73], and mice [74]. In the case of mice, deletion of only the H2A.Z-1 gene was enough for lethality, supporting the concept of a subfunctionalization of the two vertebrate variants.

Intriguingly, experiments performed by several groups in the early 2000s indicated that H2A.Z was shown to work as both a transcriptional repressor and as an activator from a broad functional perspective [75–77]. Once the dynamic interactions between the nucleosome and the basic transcriptional machinery began to be examined in further detail, it became increasingly evident that H2A.Z plays a role in poising genes for transcription where it helps to recruit RNA pol II to genes that need to be activated but is not in itself necessary for ongoing transcriptional activity [78]. As discussed further below, in many instances, activation of transcription may involve eviction of H2A.Z-containing nucleosomes near the transcription start site (TSS) of actively transcribing genes. The seemingly contradictory idea that H2A.Z functions both as an activator and a repressor of transcription was mirrored by the early structural data. Initial studies carried out with nucleosomes reconstituted *in vitro* using recombinant human H2A.Z showed a destabilization of the nucleosome resulting from H2A.Z-1–H2B dimers binding less tightly to the H3–H4 tetramer [79]. These results supported the evidence from the crystallographic data [80] which showed a destabilization of the interaction between the H2A.Z–H2B dimer and the H3–H4 tetramer. However, further biophysical characterization using native H2A.Z (consisting of a mixture of H2A.Z-1 and H2AZ-2 from chicken erythrocytes) revealed a slightly more compact nucleosome organization with similar salt dependent stability as the nucleosomes containing canonical histones [81]. For a more detailed discussion on the issues of the functional and structural controversy of H2A.Z, the reader is referred to [9, 77, 82].

**Fig. 1:**
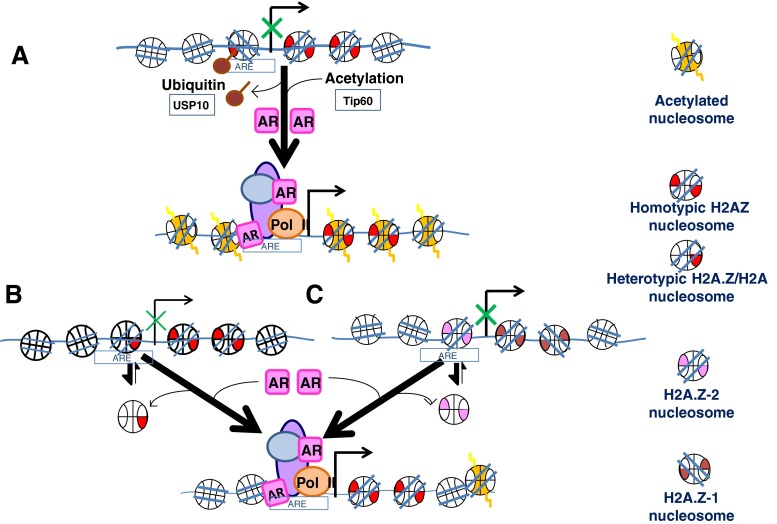
Schematic representation of the potential mechanisms involved in the displacement of H2A.Z-containing nucleosomes during transcriptional activation of androgen receptor regulated genes. **a** Histone de-ubiquitination (mediated by USP-10) [67] and histone acetylation (mediated by Tip 60 [97] are required for binding of AR to its AREs. Heterotypic H2A.Z nucleosomes at promoter regions (**b**) or H2A.Z-1 nucleosomes (**c**) may be less stable (more prone to dissociate, as indicated by the thicker arrow in the double arrow sets) and hence facilitate nucleosome removal. In this representation, H2A.Z-1 is shown in *brown* and H2A.Z-2 is in *violet*. Although shown separately here for clarity, it is possible that some of these mechanisms take place at the same time or in a combinatorial fashion. Generic histone H2A.Z (which includes the two variants) is represented in *red* and acetylated histones including H2A.Z are highlighted in *yellow dashes. AR* androgen receptor, *ARE* androgen responsive element, *Pol II* RNA polymerase II. For simplicity, histone H1 has not been shown in this representations

The explanation of the dual functional/structural role of H2A.Z has remained elusive, but several observations have been made over the years that may in themselves or in combination with one another shed some light on the problem (see Fig. 1).

### Histone posttranslational modifications

Whether H2A.Z acts as a repressor or an activator of transcription may depend on its own PTMs and on those of the histones associated with nucleosomes containing this variant. For instance, a genome-wide analysis in yeast [83], chicken [84], and several prostate cancer cell lines [68] demonstrated that histone H2A.Z acetylation is found at promoters of actively transcribing genes and nonacetylated H2A.Z is present at poised promoters of genes that are not actively undergoing transcription. Also, H2A.Z-containing nucleosomes are often enriched with marks of transcriptional activation such as tri-methylated H3K4 [85]. More recently, it has been shown that H2A.Z ubiquitination has an opposite effect [85] whereby this H2A.Z PTM is found associated with facultative heterochromatin and plays an important role in the inactivation of the human X-chromosome in female cells [85]. Both acetylation and ubiquitination marks of H2A.Z have been potentially shown to play an important role in transcriptional regulation by the AR [12, 67, 68] (Fig. 1) and deregulation of histone acetylation and histone H2A.Z composition has been shown in prostate cancer [12, 68]. From a structural perspective, histone acetylation destabilizes the nucleosome [86] and alters the binding of H2A.Z to chromatin [81], which could facilitate the eviction of H2A.Z-containing nucleosomes at promoter regions following transcriptional activation (Fig. 1).

### Homotypic and heterotypic H2A.Z nucleosomes

**Fig. 2:**
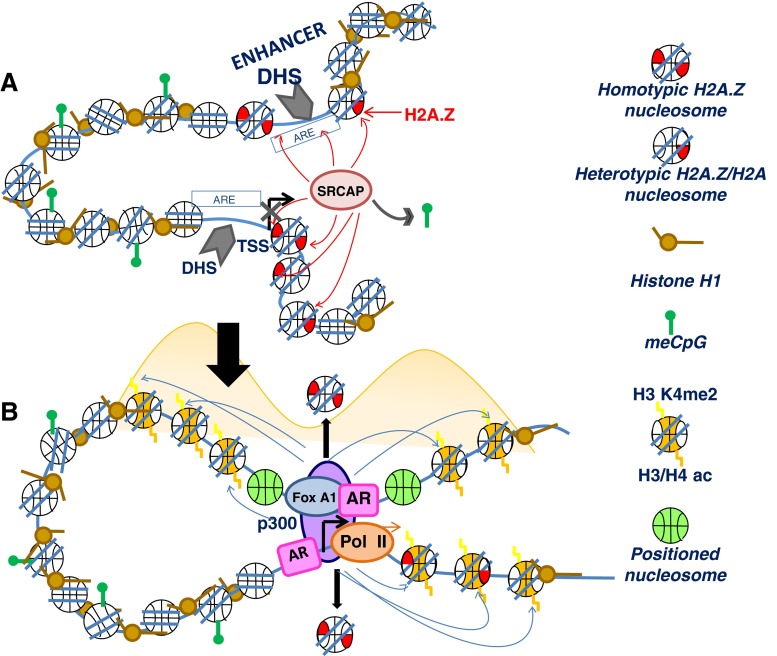
The enhancer and promoter regions come in close proximity during the process of gene activation in AR regulated genes. **a** The process involves SRCAP, a well known co-activator of AR responsible for the deposition of H2A.Z–H2B dimers into chromatin. SRCAP is an integral part of a large ATP-dependent chromatin remodeling complex which contains, among other subunits, the histone acetyltransferase p300/CBP and the forkhead protein FoxA1. The resulting complex brings RNA pol II to the transcriptional start site. Notice that in this proposed model, incorporation of H2A.Z results in histone H1 exclusion [93], demethylated DNA, and nucleosome depleted regions [106] that are responsible for the presence of DHS domains [100, 101]. **b** The activation model proposed is based on several observations from different groups and includes: presence of two positioned nucleosomes (shown in *green*) at AR regulated enhancers [102, 103] that are brought in close proximity to the promoter by chromatin looping [111], a process that likely involves FoxA1 [112]. These nucleosomes are flanked by p300/CBP acetylated chromatin domains spanning approximately 2,000 base pairs [102, 103]. The shadowed yellow waves denote the distribution of histone acetylation upon AR induction of gene expression

Each nucleosome consists of two H2A–H2B dimers. Therefore, another possibility that could account for the functional variability of H2A.Z is the presence of two H2A.Z–H2B dimers within the same (homotypic) nucleosome or the coexistence of an H2A–H2B and an H2A.Z–H2B dimer in the same (heterotypic) nucleosome (Fig. 2). Despite initial claims about the structurally unfavorable possibility of this latter situation that was based on crystallographic data [80], evidence has been provided for the existence of heterotypic H2A.Z nucleosomes in the cell [87, 88] and for the ability of these nucleosomes to be properly reconstituted *in vitro* [89–91]. Furthermore, a genome-wide analysis recently carried out in *Drosophila* showed that homotypic H2A.Z nucleosomes were enriched downstream of active gene promoters [91]. Therefore, this hints at the possibility that the functional role of H2A.Z-containing nucleosomes could be mediated by its homo- or hetero-typic H2A.Z composition based on the assumption that the latter nucleosomes are potentially less stable (Fig. 2) [91]. However, direct structural support of this claim is missing and preliminary results do not seem to support this notion.

### Presence of multiple H2A.Z variants in vertebrates

Another alternative possibility regarding the multifaceted function of H2A.Z, at least within the vertebrate lineage, could be related to the presence in these organisms of two different H2A.Z variants, H2A.Z-1, and H2A.Z-2, (Fig. 2) that have been described at the beginning of this section [10, 11]. Despite the small amino acid sequence difference between them, they exhibit a distinct electrophoretic mobility in SDS polyacrylamide gel electrophoresis, which could suggest a structural difference between them, and preliminary data indicate that they may impart the nucleosome with different structural [92] and functional [10, 12] properties. A preferential increase of H2A.Z-1 was observed in castration resistant lymph node carcinoma of the prostate (LNCaP) xenograft tumors (a form of androgen independent tumor) [12] (see below). However, a more direct experimental evidence in support of the structural and functional differences imparted by these two H2A.Z variants is still required.

### Other possibilities

The mechanisms just described may act by themselves or in a synergistic fashion. However, other factors may also be involved. For instance, H2A.Z nucleosomes are refractory to histone H1 binding [93]. While this may have little relevance in yeast which contains only reduced amounts of H1, it may be relevant within the context of mammalian cells. Also, the presence of H2A.Z affects nucleosome mobility [94] and positioning [93] *in vitro* and *in situ* [95] in a way that may be DNA sequence dependent. This has led to a model where the H2A.Z-mediated shift in nucleosome positioning at promoters may have a repressing or activating action depending on its occluding or permissive effect in allowing access to regulatory DNA sequence elements [76].

## Histone H2A.Z in androgen receptor regulated genes

The AR is a ligand-dependent transcription factor that is critical for the development of the human male phenotype and a major player in prostate cancer. Therefore, understanding the molecular chromatin determinants of its involvement in the regulation of gene expression is of critical relevance.

Several transcriptional co-activators of AR have been described to date. Some of them participate in the establishment of histone PTMs that are critical for the overall activation process. These include, HIV-1 TAT interactive protein 60 (Tip60), which acetylates histone lysines [96], as well as AR itself [97] and ubiquitin-specific protease 10 (USP-10), which specifically de-ubiquitinates H2A.Z [67]. Other activators are involved in the incorporation of histone variants into chromatin, such as SRCAP which in conjunction with Tip 60 has been shown to catalyze the ATP-dependent incorporation of H2A.Z–H2B dimers into chromatin at promoters and enhancers [5–7]. De-ubiquitination of H2A.Z by USP-10 and acetylation of the histones neighboring the AREs (by Tip60) in these regions (Fig. 1) are critical for the events that follow the activation of the gene. Another important transcriptional co-activator bromodomain-containing protein 2 (Brd2), which has two bromodomains, has been more recently identified and shown to bind to nucleosomes containing H2A.Z in a histone H4 acetylated dependent manner [98]. However, the molecular role of Brd2 in transcriptional activation remains to be elucidated.

Incorporation of H2A.Z mainly at the enhancer and proximal promoter regions of the prostate-specific antigen gene (PSA) poises the gene for activation by AR [12]. A similar situation is likely to be present at other AR regulated genes, and it is likely accompanied by DNA demethylation (Fig. 2a). DNA hypomethylation has recently been shown to be associated with a tissue-specific enhancer landscape [99]. The presence of H2A.Z in these regions may further assist in the establishment of histone H1 depleted and DNase I hypersensitive (DHS) nucleosome-free regions [100, 101] as well as in the positioning of nucleosomes at AR binding sites [102, 103]. All of this is likely to be essential for the nucleosome dynamics assisting the full assembly of the transcriptional complex containing RNA pol II (Fig. 2b). In this regard, recent literature has shown that induction of DNA hypomethylation at CpG islands of previously hypermethylated tumor suppressor genes results in nucleosome eviction [104] and an enhanced chromatin accessibility [105]. Such a process has been shown to be regulated by SRCAP-mediated H2A.Z insertion [106]. Indeed, H2A.Z is often found at promoters and enhancers which display DHS [100, 107, 108]. AR binding takes place at the AREs which are present at regulatory regions of AR regulated genes (Fig. 2), primarily at enhancers. In prostate cancer cells, androgen treatment results in the dismissal of a central nucleosome [103] which is likely responsible for the DHS associated with these regions [109] (Fig. 2). Interestingly, in prostate cancer cells, such nucleosome depleted regions appear to be present already at some of ARr enhancers in the absence of ligand in a “receptive” state for histone modifiers. Binding of androgen to AR displaces the equilibrium toward a DHS nucleosome-depleted state [110]. Further assembly of the basal transcriptional machinery complex during the activation of AR regulated genes involves the looping of chromatin [111] that brings together some of the complexes assembled at the multiple AREs that are present in the enhancers with the AREs at the promoter region (see Fig. 2b). The process appears to be mediated by forkhead box protein A1 (FoxA1) [112]. The complex brings with it p300/CBP which acetylates chromatin domains of approximately 2000 bp [102, 103], and the enhancer AREs become flanked by acetylated positioned nucleosomes [102] (Fig. 2b). This sets up the stage for the initiation of transcription.

## Altered expression of H2A.Z during prostate cancer progression

Like many other cancers, prostate cancer is the result of manifold genetic and epigenetic alterations. A recurrent theme at the genetic level involves the presence of several gene fusions [113] including a major translocation on chromosome 21 that fuses the AR regulated promoter of the transmembrane protease, serine 2 (TMPRSS2) gene to oncogenic transcription factor genes of the E-twenty six (ETS) family [114, 115]. Mutations, such as those of Fox A1, which suppress androgen signaling and promote tumor growth, also play an important role in the progression of prostate cancer to an androgen independent state [116].

On the epigenetic front, in addition to changes in the histone PTM landscape [22, 117, 118], other mechanisms involved in the progression of prostate cancer to an androgen independent state also include alterations in the DNA methylation patterns which, as with other cancer types [14, 15], constitute one of the major hallmarks of prostate cancer and its progression [119]. Both hyper- and global DNA hypomethylation have been described in the early stages and during its progression, respectively [119–121]. Interestingly, over-expression of the polycomb-group protein EZH2 (enhancer of zeste homolog 2) has also been observed [122, 123]. EZH2 is a histone-lysine N-methyltransferase that methylates lysine at position 27 of H3 and recruits DNA methyltransferases [124]. This provides a connection between some of the histone and DNA epigenetic mark alterations during the androgen-dependent stages of prostate cancer [see PEpID [43]] [123, 125–127]. Deregulation of EZH2 has been described as a mechanism for aberrant specific DNA methylation of the genes involved in cancer [128]. In prostate cancer, DNA hypermethylation is responsible for inactivation of key regulatory genes such as E-cadherin, pi-class glutathione S-transferase, and the tumor suppressors CDKN2, PTEN, and IGF-II [129].

Interestingly, an anticorrelation has been observed between DNA methylation and the occurrence of H2A.Z in the genome, such that genomic regions that are enriched in DNA methylation are often devoid of histone H2A.Z [130, 131]. Such correlations have implications for the chromatin environment of the regions associated with AR.

Given the alterations in DNA methylation during the progression of prostate cancer [119], and the antagonistic relationship between DNA methylation and H2A.Z occupancy, it is not surprising that a few recent papers have also implicated the histone variant H2A.Z in this type of cancer [8, 12, 67, 68, 98]. Furthermore, inhibition of SRCAP expression has been shown to interfere with the androgen-dependent stages of prostate cancer cell growth [8]. As was seen with the expression of the PSA gene [12], it was found that in prostate cancer cells there is an H2A.Z reorganization that poises the oncogene promoters for activation. The overall levels of H2A.Z decrease at the TSSs of such promoters upon activation which is accompanied by a gain of acetylated H2A.Z [68]. In this way, H2A.Z operates as a facilitator of transcription that is evicted or subject to a rapid dynamic turnover once the gene is undergoing cycles of transcription in quick succession in the presence of androgen [12].

**Fig. 3:**
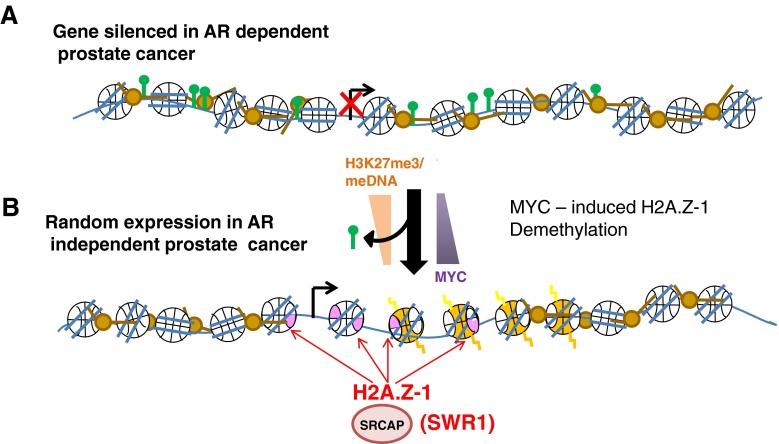
A potential mechanism to explain the androgen-independent gene expression program (**a**). An inactive gene in androgen-dependent prostate cancer could be randomly activated in the androgen-independent stages of cancer progression (**b**). SRCAP would be involved in the deposition of histone H2A.Z–H2B dimers resulting from the MYC-induced over-expression of H2A.Z-1 in androgen-independent prostate cancer cells [12]. As H2A.Z and DNA methylation have been shown to have an antagonistic genomic distribution [130, 144] and gene occupancy during B-cell lymphomagenesis [131], increased H2A.Z-1 deposition would, in a similar fashion, exacerbate the global DNA hypomethylation that has been shown to be a feature of late-stage metastatic prostate cancer [134]

Histone H2A.Z appears to have several important roles during the androgen independent stages of prostate cancer as well. A significant increase in H2A.Z, mainly affecting the H2A.Z.1 subtype, was observed in castration resistant LNCaP xenograft tumors (a form of androgen-independent tumor) [12]. Interestingly, the promoters of H2A.Z.1 and H2A.Z.2 are completely different where that of H2A.Z-1 contains several myelocytomatosis viral oncogene (MYC) regulatory elements. These elements are likely responsible for the increase in H2A.Z-1 expression observed in castration-resistant tumors [12]. Indeed, the increasing levels of MYC in castration-resistant prostate cancer [132] result in a global loss of H3K27me3 [133] (Fig. 3) and hence a global decrease in DNA methylation which is a characteristic feature of late- or end-stage metastatic prostate cancer [134].

It is not possible to know whether the increase in H2A.Z-1 observed by Dryhurst et al.[12] in the transition to castration resistant prostate cancer is required to mediate a specific cellular function during the transition to androgen independence or if it is simply a by-product of a more aggressive and advanced stage of cancer. As pointed out in [12], two possibilities exist (Fig. 3).

We have already described how H3K27me3 is important for the regulation of the levels (both specific and global) of DNA methylation. A negative correlation has been found between the aggressiveness of prostate cancer (Gleason score and pathological state) and the global loss of H3K27me3 which is linked to MYC over-expression [133, 135] (Fig. 3). Such an increase in MYC could also affect the activation of the MYC-responsive H2A.Z.1 promoter. This would lead to an over-expression of this histone variant which, given its antagonistic relationship with DNA methylation, could in turn exacerbate the global DNA hypomethylation [12].

An alternative possibility could be that the increased expression of H2A.Z-1 would lead to an altered association of this histone variant with chromatin which could increase the plasticity of castration resistant cancer cells. Indeed, an AR responsiveness persists in the androgen-dependent to -independent transition of prostate cancer [136] and, with its co-activator SRCAP, AR could potentially bring the over-expressed H2A.Z.1 to different promoters. As H2A.Z is highly enriched within the promoter regions of transcriptionally active genes [108] such an alteration could potentially lead to a completely altered program of gene expression (Fig. 3b). Notably, it has been shown that AR induces a distinct transcriptional program in androgen-independent prostate cancer than in androgen-dependent prostate cancer [137, 138].

## Concluding remarks and future perspectives

From all the above, H2A.Z appears to be an important player in the regulation of AR regulated genes which has manifold roles in the regulation of the structural chromatin transitions at the base of the native and deregulated condition of such genes. Hence, the involvement of H2A.Z in prostate cancer is not surprising. Its active participation in the androgen-dependent stages of the disease is clearly reflected by the dependence on SRCAP for cell proliferation [8]. In contrast, EZH2-mediated DNA methylation of tumor suppressor genes at these early stages may simply prevent H2A.Z from binding and activation of such genes. In the androgen-independent stages of this cancer, the preferential binding of H2A.Z-2 to H3K4me3 [12] seems to play an active role in directing AR to the M-phase cell cycle genes [137, 139]. While this again suggests a causative involvement, the connection between the further MYC-dependent DNA hypomethylation and over-expression of H2A.Z-1, which is also observed in the later androgen-independent stages, remains to be determined. It is nevertheless clear that whatever the causative and consequential nature of the effects elicited by histone variant are, they are closely intertwined and critically important for prostate cancer development and its progression.

Other examples of histone variant involvement in cancer have also been described [56]. For instance, hisone H2A.Z has also been shown to be involved in the regulation of ER-dependent genes in breast cancer [53, 66, 95, 140, 141]; macroH2A has a tumor suppressive function [64, 142, 143]. Nevertheless the literature available is scarce, and it has not been until very recently that histone variants have started being perceived as “emerging players in cancer biology” [13].
